# The potential of Nutri-Score to discriminate foods according to environmental impact

**DOI:** 10.1007/s00394-025-03635-8

**Published:** 2025-03-12

**Authors:** Elly Steenbergen, Reina E. Vellinga, Joline WJ Beulens, Elisabeth HM Temme

**Affiliations:** 1 National Institute for Public Health and the Environment, Bilthoven, The Netherlands; 2 Department of Epidemiology & Data Science, Amsterdam UMC, location Vrije Universiteit, Amsterdam, The Netherlands; 3 Amsterdam Public Health research institute, Amsterdam, The Netherlands

**Keywords:** Nutri-score, Front-of-pack label, Environmental impact, Sustainability, Planetary health, Human health

## Abstract

**Purpose:**

Nutri-Score aims to aid consumers in making informed food choices based on nutritional quality. To guide consumers regarding both the nutritional quality and environmental impact of foods, it would be beneficial when Nutri-Score could also discriminate foods by environmental impact. This study investigated the association of Nutri-Score with the environmental impact indicators greenhouse gas (GHG) emissions and blue water consumption.

**Methods:**

Nutri-Score classifications were calculated for generic foods of the Dutch Food Composition Database, combined with GHG emissions (kg CO_2_ equivalents) and blue water consumption (m^3^) of foods using the Dutch Life Cycle Assessment Food database (*n* = 1,611). Spearman correlations were calculated between Nutri-Score (expressed as the numerical final score and as points for algorithm components) and the environmental impact indicators. This was performed by Nutri-Score algorithm (i.e. general foods, added fats, nuts and seeds, and beverages) and food group. Selected food groups were studied further.

**Results:**

Nutri-Score (final score) correlated significantly with GHG emissions for general foods (*r* = 0.29), added fats, nuts and seeds (*r* = 0.25), and negatively with blue water consumption for added fats, nuts and seeds (*r*=-0.51). Saturated fatty acids and protein were significantly correlated with GHG emissions (*r* = 0.52 and *r* = 0.43) for general foods, and some general food groups with GHG emissions and blue water consumption, namely bread (*r* = 0.60 and *r* = 0.53) and savoury sauces (*r* = 0.52 and *r* = 0.53).

**Conclusion:**

Currently, Nutri-Score’s ability to discriminate foods according to environmental impact is limited. To use Front-of-Pack labeling to guide consumers on both nutritional quality and environmental impact, exploration of revisions to Nutri-Score or the use of additional tools is needed.

**Supplementary Information:**

The online version contains supplementary material available at 10.1007/s00394-025-03635-8.

## Introduction

Food choices are of major importance because of the impact on health and the environment. Unhealthy diets, characterized as being high in unfavourable components (energy, salt, sugar, saturated fatty acids, red and processed meat) and low in favourable components (fruits, vegetables, and legumes), are recognized as a major preventable risk factor for non-communicable diseases (NCDs) such as cardiovascular diseases and type II diabetes, and premature deaths [1]. Furthermore, current food production and consumption practices are a significant contributor to environmental change, accounting for 26–30% of global greenhouse gas (GHG) emissions [2]. In general, animal-based foods such as (processed) meat, dairy and cheese are major contributors to the saturated fatty acids and salt intake and generally have more impact on the environment than plant-based foods. Recent evidence shows that these less environmentally sustainable foods are generally associated with a higher risk for negative health outcomes, such as type II diabetes and heart disease [3]. In contrast, plant-based foods are major sources of dietary fiber, plant-based protein and essential nutrients, which benefit human health, and are associated with a lower environmental impact [3–6]. Therefore, changing dietary patterns towards more plant-based patterns has the potential to simultaneously reduce the prevalence of NCDs and mitigate environmental impact [7–9].

Changing dietary patterns by promoting both healthier and more environmentally sustainable food choices among consumers is a present-day challenge [10]. The Farm-to-Fork strategy by the European Commission strives to develop a more uniform food labelling system, harmonizing the health and environmental impact aspects of food choices [11]. Recently, the European Parliament and the council of the European Union updated their directive for the green transition, including concerns on labelling such as transparency and credibility [12]. Front-of-Pack (FOP) nutritional labels are a policy tool to help consumers make informed food choices based on the nutritional quality of a food. FOP environmental impact labels inform about the environmental impact of a food. FOP labels may also stimulate food reformulation by food manufacturers. Nutri-Score has been implemented as the official FOP nutritional label in several European countries including the Netherlands [13]. Nutri-Score is a five-colour and letter-coded label with classifications ranging from dark green A to dark orange E, and aims to help consumers in making the healthy food choice more easily. It is calculated with three across-the-board algorithms (for the categories general foods, added fats, nuts and seeds, and beverages) based on nutrient contents as provided by the food label information. Nutri-Score differentiates between healthier and less healthy choices and can be used by consumers for comparisons at a glance. Nutri-Score has been extensively studied and was found to be effective in the discrimination between healthier and less healthy foods in several European countries [14–21].

The Nutri-Score algorithm does not directly take into account aspects regarding the environmental impact of the foods as Nutri-Score was not initially intended and designed to inform consumers in making environmentally sustainable food choices [22]. However, several characteristics of Nutri-Score may also allow for an evaluation of the environmental aspects of the foods, such as the generally known associations of animal and plant-based foods with higher and lower GHG emissions respectively. These are, to a certain extent, reflected in dietary guidelines that recommend to consume more plant-based foods and less animal-based foods, for example in the Dutch food-based dietary guidelines [23], which is also reflected by Nutri-Score. With Nutri-Score, foods with a higher content of vegetables, fruits and legumes receive more favourable classifications, and saturated fatty acids content (mainly occurring in animal-based products) is one of the unfavorable components. In addition, one of the changes in the updated Nutri-Score algorithm which could potentially improve the alignment of Nutri-Score with environmental targets involves a maximum number of points allocated to protein content for red meat. As the interest in environmental impact of foods is increasing amongst consumers and Nutri-Score is increasingly implemented in countries as the FOP label on packaged foods, it would be beneficial if Nutri-Score could guide consumers on both the nutritional quality and environmental impact of foods.

A recent study showed that, in a virtual setting using a Nutri-Score based tax, setting higher retail prices for less healthy foods with Nutri-Scores D and E resulted in lower GHG emissions and land use of food purchases [24]. However, direct associations between Nutri-Score and environmental impact indicators of foods have not been studied yet. For example, it is not known whether foods with a more favourable nutritional quality (Nutri-Score classifications A-B, e.g. fruits and vegetables) are associated with a lower environmental impact, compared to whether foods with less favourable nutritional quality (Nutri-Score classifications D-E, e.g. processed meat) are associated with a higher environmental impact. Additionally, it is not known whether differentiation of environmental impact in Nutri-Score classifications (i.e. distribution over several classifications) occurs within food groups. Food composition data and environmental impact data of generic foods, also categorized into food groups, can be used for exploratory analyses on the ability of a FOP label to inform about both the nutritional quality and the environmental sustainability. Therefore, to study Nutri-Score’s potential to discriminate foods according to environmental impact, the aim of the present study is to investigate the association of Nutri-Score with environmental impact indicators per kg of foods using information on generic foods.

## Materials and methods

### Data Preparation

Food composition data of generic foods were available from the Dutch Food Composition Database (NEVO online version 2021/7.1) [25]. This database contains, amongst others, data on the energy, salt, sugar, saturated fatty acids, protein, and fiber content per 100 gram of foods [26]. The environmental impact of generic foods were available from the Dutch Life Cycle Assessment (LCA) Food database [27], expressed per kg of food, including data on the following mid-point indicators: GHG emissions in kg CO_2_ equivalents (eq), land use (m^2^a crop eq), terrestrial acidification (kg SO_2_ eq), freshwater eutrophication (kg P eq), marine eutrophication (kg N eq), and blue water consumption (m^3^, total amount of surface or groundwater that is used, transferred, evaporated or disposed). The functional unit is 1 kg of prepared food or drink at plate. More detailed information on the LCA data can be found elsewhere [28]. Except for blue water consumption, the indicator GHG emissions is highly correlated with other environmental impact indicators from the database, as found in a previous study [28]. Therefore, for the purpose of the present study, only GHG emissions and blue water consumption were included as environmental impact indicators.

Nutri-Score’s underlying algorithm takes into account the overall nutritional quality of a food by allocating points to favourable (total protein, fiber, fruits, vegetables, and legumes content) and unfavourable components (total energy, salt, sugar, and saturated fatty acids content) [29]. Recently, the Nutri-Score algorithm has been updated, including improvements of the algorithm based on scientific literature and the synergy with country specific dietary guidelines [22, 30]. The Nutri-Score algorithms are developed for three main categories: general foods, added fats, nuts and seeds, and beverages. Within these three categories, slightly different calculation rules apply for certain foods, such as red meat, cheese, and beverages that contain specific non-nutritive sweeteners. The Nutri-Score algorithm calculates a final score (numerical variable) based on points allocated to different components (per 100 g) which is then translated into a classification ranging from dark green A to dark orange E (categorical variable). In the present study, Nutri-Score calculations were based on the updated algorithm and according to the guidelines (version dated 24th of November 2023) [29]. In order to calculate the Nutri-Score classifications of foods, some assumptions had to be made as not all information was available from the databases. Firstly, based on the NEVO name, a meat product was assigned to contain red meat or not. Secondly, when the NEVO name of a beverage contained the terms ‘light’, ‘zero’, ‘sweeteners’ or ‘sugar free’, the assumption was made that it contained non-nutritive sweeteners to which Nutri-Score allocates negative points. For foods, data on the fruits, vegetables and legumes content (%) were not available from the NEVO database. These were estimated at food group level (Supplementary material Table S1).

Food composition data were matched with environmental impact data based on NEVO code (*n* = 2,626). Extrapolations of primary environmental impact data were performed for NEVO codes for which primary environmental impact data were not available [23, 28]. Foods with missing values for either the nutrient contents needed for the calculations of the Nutri-Score, or the environmental impact indicators, were excluded from the analyses (*n* = 927). Food groups consisting of less than 10 food items (such as mixed dishes and miscellaneous foods) were excluded as these were considered too small for the interpretation of results. These food groups were excluded from all analyses in order to describe and interpret results with the same dataset. In addition, food groups to which Nutri-Score is not applicable (such as alcoholic beverages and foods for special nutritional use, *n* = 88) were excluded from the analyses.

### Data analyses

The distribution of Nutri-Score (final score and classifications A to E) as well as the distributions of GHG emissions (in kg CO_2_ equivalents) and blue water consumption (in m^3^) were calculated by category of the Nutri-Score algorithm and by food group.

To study the relationship between Nutri-Score and the environmental impact of foods, the environmental impact indicators were initially tested for normality by the Shapiro-Wilk (*n* ≤ 50) and Kolmogorov-Smirnov (*n* > 50) test at food group level. *P*-values of results were lower than 0.05 for both environmental impact indicators for each food group. Therefore, correlation analyses were carried out based on the non-parametric Spearman’s correlation test to explore the relationship between Nutri-Score and the environmental impact indicators. A correlation was considered significant when *p* < 0.05.

First, Spearman correlations were calculated for the final score of Nutri-Score and the number of points allocated to Nutri-Score algorithm components with the environmental impact indicators by category of Nutri-Score algorithm. Additionally, Spearman correlations were calculated between the final score of Nutri-Score and environmental impact indicators by food group. Food groups with significant correlations between the final score Nutri-Score and environmental impact indicator and relevant food groups were further studied by calculating and plotting the median environmental impact per Nutri-Score classification by food group. Analyses in the present study were carried out using SAS (version 9.4) and R (version 4.3.0).

## Results

In total, 1611 foods with food composition data were combined with environmental impact data.

**Fig. 1:**
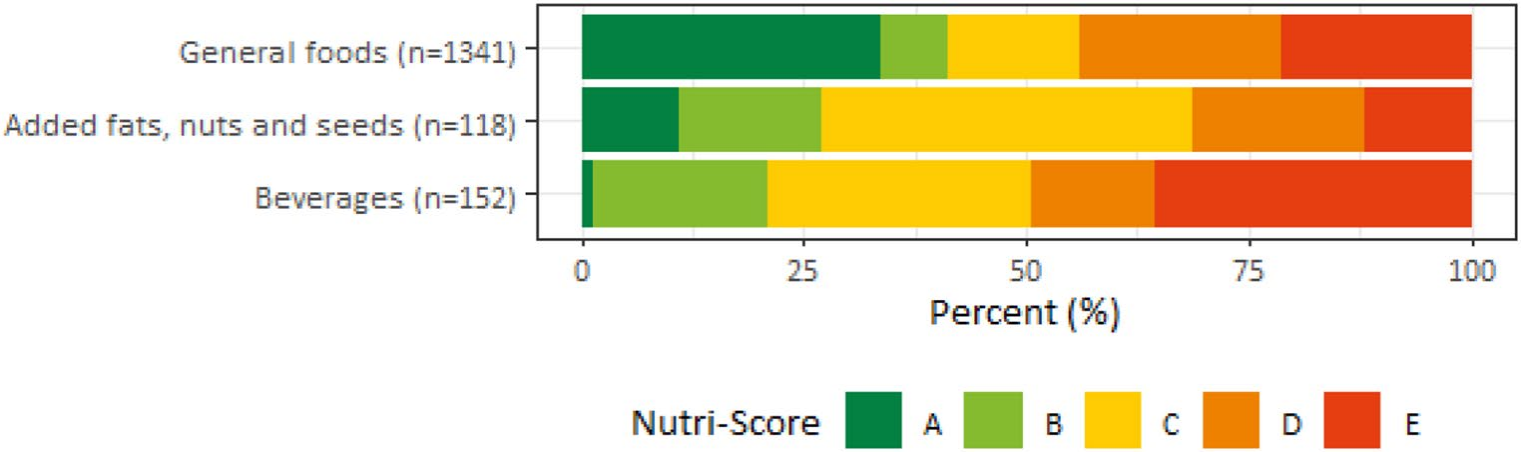
Distribution of Nutri-Score classifications (A-E, %) of foods by category of Nutri-Score algorithm

**Fig. 2:**
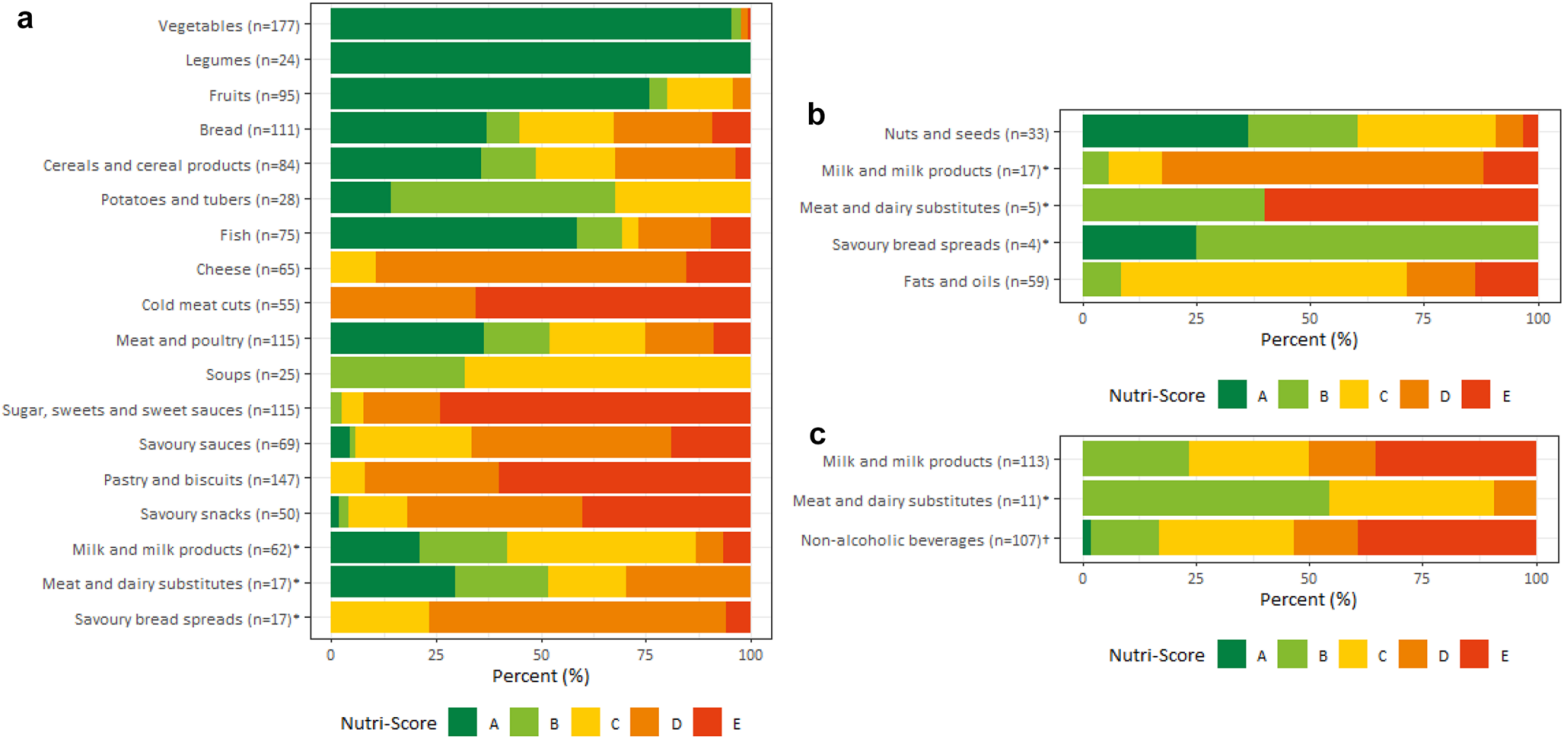
(**a**) Distribution of Nutri-Score classifications (A-E, %) of foods by food group within Nutri-Score category general foods. *only includes the foods calculated with the algorithm for general foods. (**b**) Distribution of Nutri-Score classifications (A-E, %) of foods by food group within Nutri-Score category added fats, nuts and seeds. *only includes the foods calculated with the algorithm for added fats, nuts and seeds. (**c**) Distribution of Nutri-Score classifications (A-E, %) of foods by food group within Nutri-Score category beverages. *only includes the foods calculated with the algorithm for beverages, †within Nutri-Score category beverages, only mineral waters are able to receive a Nutri-Score classification A

The distribution of Nutri-Score classifications and final score of Nutri-Score were calculated by category of Nutri-Score algorithm and by food groups for each category of Nutri-Score algorithm (Figs. 1 and 2; Supplementary material Table S2-S3). Within most food groups, a range of three to five Nutri-Score classifications were present. For vegetables and legumes, the Nutri-Score classifications were (mostly) A, whereas cold meat cuts were classified as either D or E, and soups as either B or C. Nutri-Score classifications D and E were largely present within the food groups cheese, sugar, sweets and sweet sauces, pastry and biscuits, and savoury snacks.

**Fig. 3:**
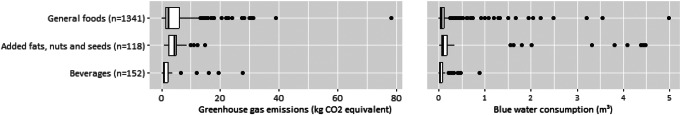
Distribution of greenhouse gas emissions (kg CO_2_ equivalents) and blue water consumption (m^3^) per kg of food by category of Nutri-Score algorithm. The white box represents the interquartile range, the black vertical line in the box represents the median of the data, the whiskers of the box present the minimum and maximum values of the data, excluding the outliers, which are the black dots. The specific outlier within general foods for greenhouse gas emissions is dried goji berries

**Fig. 4:**
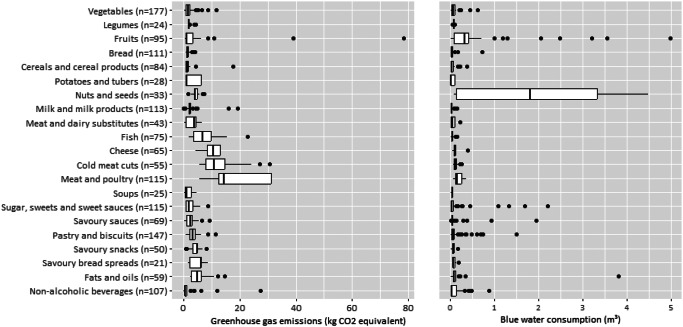
Distribution of greenhouse gas emissions (kg CO_2_ equivalents) and blue water consumption (m^3^) per kg of food by food group. The white box represents the interquartile range, the black vertical line in the box represents the median of the data, the whiskers of the box present the minimum and maximum values of the data, excluding the outliers, which are the black dots. The specific outlier within general foods for greenhouse gas emissions is dried goji berries

The distribution of GHG emissions and blue water consumption by category of Nutri-Score algorithm and by NEVO food groups are shown in Table 1; Figs. 3 and 4 and Supplementary material Table S4 and Figures S1-S4. GHG emissions were higher in the Nutri-Score category general foods and added fats, nuts and seeds, rather than beverages. Especially animal-based foods such as fish, cheese, cold meat cuts, and meat and poultry show relatively high medians on GHG emissions (6.95–14.4 kg CO2 equivalents per kg of food). For blue water consumption, the food groups fruits, nuts and seeds, and meat and poultry have relatively high medians (0.08–0.13 m³ per kg of food). Compared to the Nutri-Score categories general foods and beverages, the category added fats, nuts and seeds is high in median blue water consumption (0.10 vs. 0.05 and 0.02 m³ per kg of food respectively), largely due to the blue water consumption associated with the food group nuts and seeds.

**Table 1:** Distribution of greenhouse gas emissions (kg CO_2_ equivalents) and blue water consumption (m^3^) per kg of food by food group

	Greenhouse gas emissions (kg CO_2_ equivalents)							Blue water consumption (m^3^)					
Food group	N	Mean	P5	P25	P50	P75	P95	Mean	P5	P25	P50	P75	P95
Vegetables	177	1.80	0.40	0.78	1.56	2.23	4.50	0.07	0.01	0.03	0.05	0.09	0.20
Legumes	24	2.34	1.93	1.93	1.93	2.07	3.73	0.07	0.04	0.07	0.07	0.07	0.07
Fruits	95	3.56	0.53	0.93	1.21	3.44	6.41	0.44	0.02	0.08	0.31	0.41	2.04
Bread	111	1.57	1.03	1.19	1.44	1.77	2.94	0.06	0.02	0.02	0.02	0.04	0.17
Cereals and cereal products	84	1.67	0.88	0.88	1.52	1.71	2.42	0.05	0.01	0.01	0.02	0.06	0.17
Potatoes and tubers	28	3.25	0.92	0.92	1.09	6.15	6.15	0.04	0.01	0.01	0.01	0.09	0.09
Nuts and seeds	33	4.20	1.67	3.89	4.06	4.87	7.35	1.96	0.10	0.13	1.80	3.31	4.44
Milk and milk products	113	2.82	1.34	2.03	2.19	2.52	4.72	0.03	0.01	0.02	0.02	0.03	0.04
Meat and dairy substitutes	43	2.83	0.26	0.76	3.73	4.44	5.26	0.06	0.01	0.01	0.04	0.10	0.11
Fish	75	7.94	2.17	3.58	6.95	9.89	15.4	0.04	0.02	0.02	0.04	0.05	0.11
Cheese	65	9.96	4.61	8.50	10.7	13.1	13.1	0.09	0.04	0.07	0.09	0.10	0.12
Cold meat cuts	55	12.7	5.73	7.94	10.8	14.7	27.3	0.12	0.06	0.08	0.11	0.13	0.22
Meat and poultry	115	19.7	8.54	12.4	14.4	31.1	31.3	0.19	0.08	0.12	0.15	0.25	0.36
Soups	25	2.08	0.31	0.71	1.15	2.82	4.53	0.03	0.01	0.03	0.04	0.05	0.05
Sugar, sweets and sweet sauces	115	2.46	0.84	0.84	1.89	3.48	6.06	0.11	0.01	0.01	0.03	0.06	0.27
Savoury sauces	69	2.76	0.43	1.17	2.48	2.92	6.44	0.09	0.01	0.04	0.05	0.05	0.28
Pastry and biscuits	147	3.41	1.52	2.29	3.27	4.00	5.80	0.12	0.02	0.03	0.06	0.08	0.60
Savoury snacks	50	4.25	1.07	3.38	4.83	4.83	8.11	0.07	0.02	0.05	0.07	0.08	0.16
Savoury bread spreads	21	5.15	1.67	2.14	6.32	6.32	8.68	0.08	0.04	0.05	0.05	0.09	0.17
Fats and oils	59	5.77	2.52	2.52	5.01	6.17	12.2	0.15	0.02	0.05	0.08	0.11	0.34
Non-alcoholic beverages	107	1.52	0.36	0.60	0.71	1.10	3.61	0.09	0.01	0.01	0.02	0.12	0.45

**Fig. 5:**
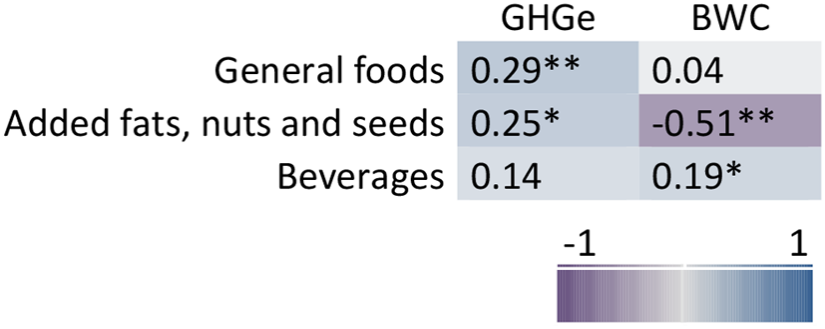
Heat map of Spearman’s correlation between final score of Nutri-Score and environmental impact indicators by category of Nutri-Score algorithm. GHGe: greenhouse gas emissions (kg CO_2_ equivalents), BWC: blue water consumption (m^3^), *p < 0.05, **p < 0.001

**Fig. 6:**
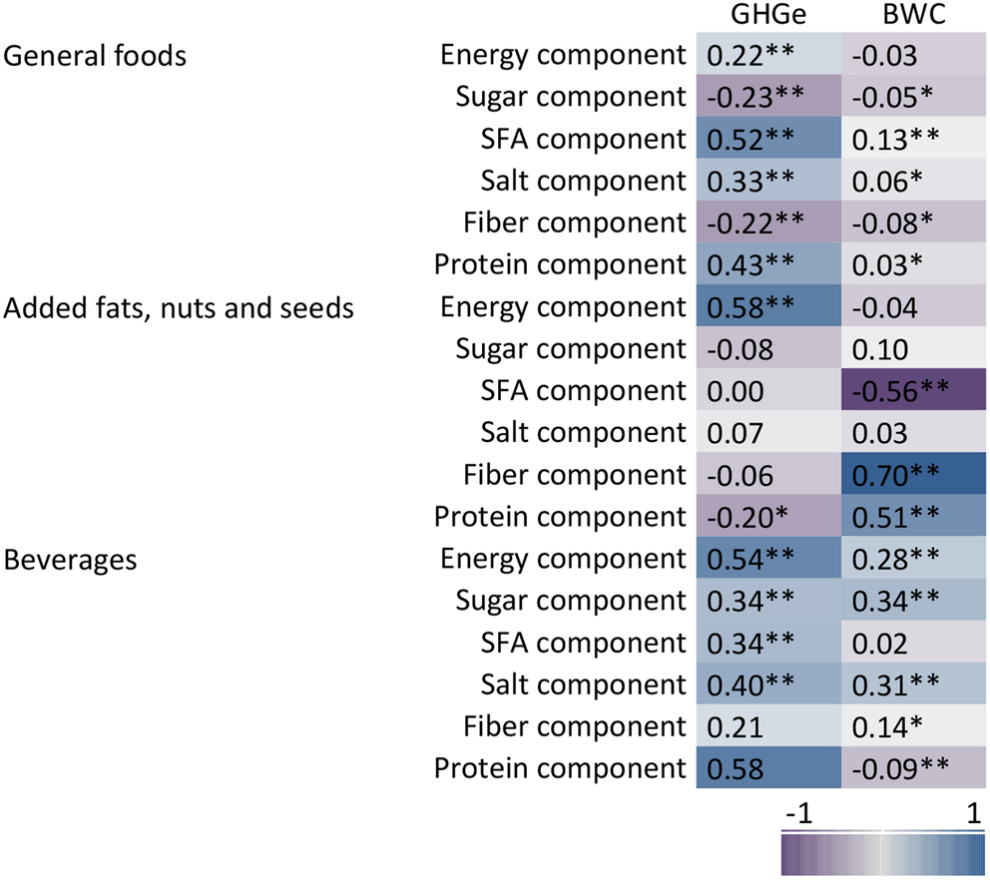
Heat map of Spearman’s correlation between algorithm component points and environmental impact indicators by category of Nutri-Score algorithm. GHGe: greenhouse gas emissions (kg CO_2_ equivalents), BWC: blue water consumption (m^3^), SFA: saturated fatty acids, *p < 0.05, **p < 0.001

At the level of category of Nutri-Score algorithm, the final score of Nutri-Score was correlated significantly and positively with GHG emissions for general foods (*r* = 0.29) and added fats, nuts and seeds (*r* = 0.25), indicating that a higher final score (in the direction of Nutri-Score classification E) has a higher environmental impact (Fig. 5). In contrast, for the Nutri-Score category added fats, nuts and seeds, the final score was significantly and negatively correlated (*r*=-0.51) with the indicator blue water consumption, which indicates that a higher final score had a lower blue water consumption. Nutri-Score classifications of beverages were significantly and positively correlated with blue water consumption (*r* = 0.19). Looking at the correlations with the points for algorithm components (Fig. 6, Supplementary material Table S5), stronger, positive correlations were found between the points allocated to the saturated fatty acids and protein components and GHG emissions within the general foods (*r* = 0.52 and *r* = 0.43 respectively). Significant correlations were found for the saturated fatty acids (*r*=-0.56), fiber (*r* = 0.70) and protein (*r* = 0.51) components and blue water consumption for the added fats, nuts and seeds. For added fats, nuts and seeds and beverages, the points allocated to the energy component were positively correlated (*r* = 0.58 and *r* = 0.54 respectively) with GHG emissions.

**Fig. 7:**
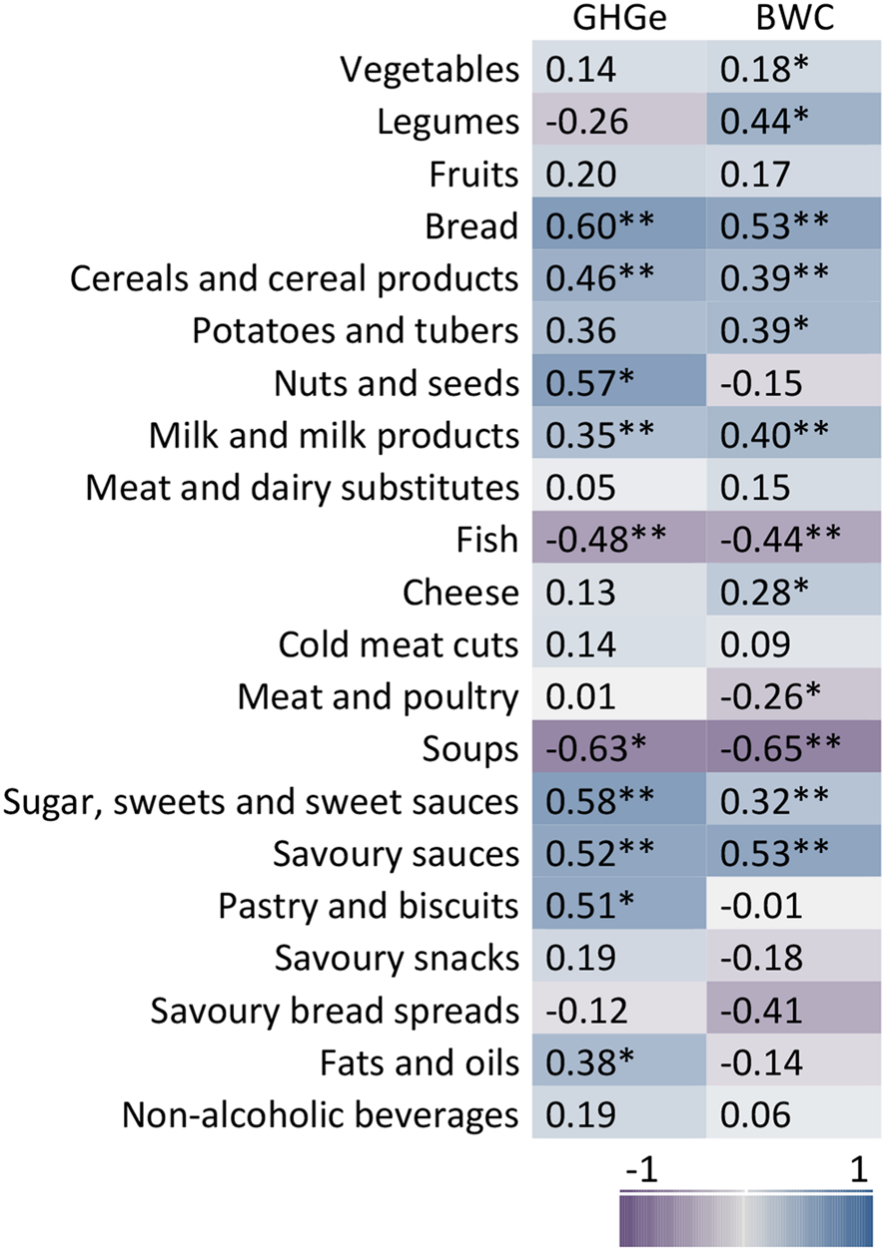
Heat map of Spearman’s correlation between final score Nutri-Score and environmental impact indicators by food group. GHGe: greenhouse gas emissions (kg CO_2_ equivalents), BWC: blue water consumption (m^3^), *p < 0.05, **p < 0.001

At food group level, moderate but significant correlations were found for some food groups between the final score of Nutri-Score and either the indicator GHG emissions or blue water consumption (Fig. 7, Supplementary material Table S6). Among the animal-based food groups, milk and milk products had the highest significant positive correlation between the final score of Nutri-Score and environmental impact indicators (*r* = 0.35 for GHG emissions and *r* = 0.40 for blue water consumption). For other animal-based food groups as well as the meat and dairy substitutes, correlations with final score of Nutri-Score and GHG emissions were not significant except for fish, which was inversely and significantly correlated (*r*=-0.48). For both GHG emissions and blue water consumption, bread (*r* = 0.60 and *r* = 0.53 respectively) and savoury sauces (*r* = 0.52 and *r* = 0.53 respectively) were strongly and positively correlated, whereas soups (*r* = -0.63 and *r* = -0.65 respectively) were strongly and negatively correlated. For legumes, nuts and seeds, sugar, sweets and sweet sauces, and pastry and biscuits, either GHG emissions or blue water consumption were relatively stronger correlated, ranging between *r* = 0.44 and *r* = 0.58.

**Fig. 8:**
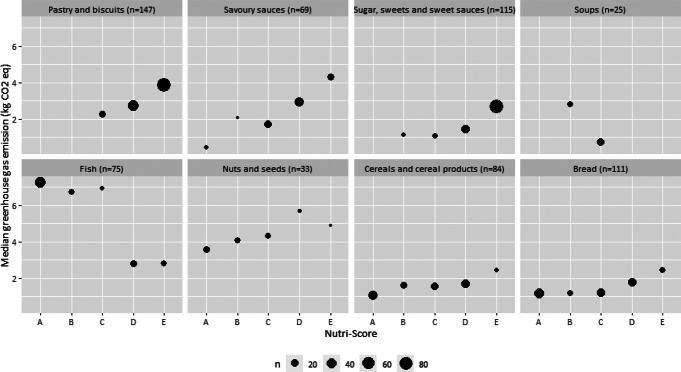
Median greenhouse gas emissions (kg CO_2_ equivalents) within Nutri-Score classifications by food group. The size of the dot is proportional to the number of observations

**Fig. 9:**
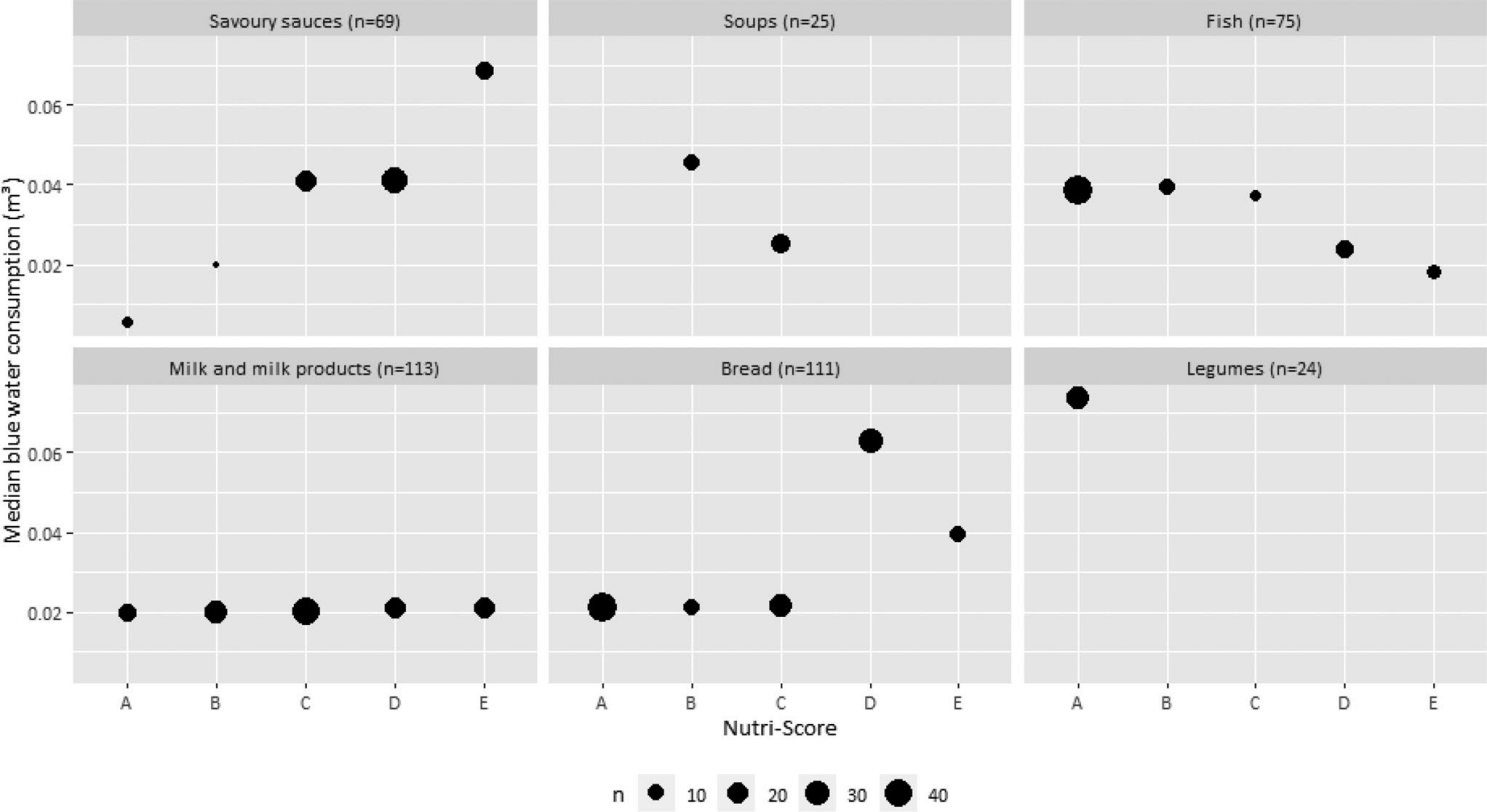
Median blue water consumption (m³) within Nutri-Score classifications by food group. The size of the dot is proportional to the number of observations

Figures 8 and 9 and Supplementary material Table S7 show the median GHG emissions and median blue water consumption within Nutri-Score classifications and according to the number of foods by food group. Overall, the medians of the environmental impact indicators within Nutri-Score classifications reflect the significant correlations observed for the food groups (Fig. 7, Supplementary material Table S6). This was visibly reflected by the median environmental impact indicators for each Nutri-Score classification by a relatively low median impact for Nutri-Score classification A towards relatively higher median impact for Nutri-Score classification E. For fish and soups, the opposite was observed as there was a negative correlation between final score of Nutri-Score and the environmental impact indicators.

## Discussion

The present study aimed to investigate whether the FOP nutritional label Nutri-Score is associated with environmental impact indicators. Significant correlations with either one or both of the environmental impact indicators (i.e. GHG emissions and blue water consumption) were found between food groups. These correlations were in terms of categories of Nutri-Score algorithm (e.g. general foods, added fats, nuts and seeds) with the final score of Nutri-Score as well as with the points allocated by Nutri-Score algorithm components (e.g. energy, saturated fatty acids, and protein content depending on the category of Nutri-Score algorithm). These correlations were often moderate and also found for both environmental impact indicators within food groups. The results found in the present study indicate that for some food groups such as bread and savoury sauces, healthier food choices (more favourable Nutri-Score) also benefit planetary health (lower environmental impact).

Correlations that were observed between food groups and the environmental impact indicators are in line with the generally known association between animal-based foods and environmental impact as saturated fatty acids and protein content (both of which are relatively high in animal-based foods) of general foods were positively stronger correlated with GHG emissions. At food group level, animal-based foods, such as milk and milk products, cheese, and meat and poultry had relatively high GHG emissions compared to other food groups such as healthier, plant-based foods. These findings are similar to what was presented by Poore and Nemecek (2018), as meat and dairy had the highest emissions and whereas plant-based alternatives had lower GHG emissions [31].

In the present study, while it was observed that animal-based foods had relatively high GHG emissions, heterogeneous results were found for associations between the final score of Nutri-Score of food groups and the environmental impact indicators. For instance, the final score of Nutri-Score of milk and milk products were indeed significantly and positively correlated with both environmental impact indicators. Cold meat cuts, however, showed no significant correlations for both indicators, whereas cheese and meat and poultry were moderately correlated (positively and negatively respectively) with blue water consumption. While being relatively high in GHG emissions, cheese had a relatively small spread in the distributions of GHG emissions. This may be due to the use of extrapolated data, which may have led to the small and insignificant correlation as measured in this study. Another explanation for the small and insignificant correlation could be the low variation in the nutritional quality of foods within the food group as only two and three Nutri-Score classifications were covered by the distribution for cheese and cold meat cuts respectively. Meat and poultry, however, were distributed over all Nutri-Score classifications. Soups also covered only two Nutri-Score classifications (B and C) and soups that contain meat had higher environmental impact and yet did not necessarily receive a more unfavourable Nutri-Score. This could be explained by the type of foods within food groups in the dataset that was used for this study. For example, the negative correlations that were found for fish and soups most likely resulted due to the majority of processed fish with additions (e.g. heating processes such as smoking, and tinning or addition of sauce and oil) and soups that contain meat. The unprocessed or raw fish were relatively underrepresented in the dataset. It may be that more processed, unhealthier foods have lower environmental impact due to the addition of more cost-effective ingredients, resulting in an overall lower environmental impact of the food, which is not reflected by the Nutri-Score classification.

The findings in the present study are comparable with previous findings from Italy by Muzzioli et al. (2023), in which Nutri-Score classifications on food group level were low or moderately correlated with environmental impact indicators. However, these were calculated with the previously used Nutri-Score algorithm and only the correlations of two food groups (i.e. meats, and oils and fats) with GHG emissions could be compared with the results in the present study. It was considered that these low-to-modest correlations were due to the calculation of Nutri-Score classifications based on 100 g of food rather than reference amounts as reflected by consumption in dietary patterns [32]. This was also the case in the present study.

Other studies have investigated the impact of FOP nutritional and/or environmental labels by consumers. As studied in a virtual supermarket in France, using a FOP environmental label which resembles the Nutri-Score (i.e. classifications from dark green A to dark orange E) was found to reduce the environmental impact of food choices (-0.17 millipoint per kg of food) due to changes of food choices between food groups (i.e. from meat to vegetarian meals) [33]. Similar results were found in a virtual supermarket in the United Kingdom, where the environmental impact of food choices was significantly reduced when a FOP environmental label was displayed alone (mean difference of -1.3 in environmental impact score) or together with Nutri-Score (mean difference of -2.0 in environmental impact score) [34]. These findings indicate that a FOP environmental label alongside a FOP nutritional label such as Nutri-Score, can be effectively used to aid consumers in making environmentally sustainable food choices. However, opposite results were found in a study from Belgium, in which displaying both Nutri-Score and Eco-Score on foods in a virtual supermarket did not improve the environmental impact of food choices, whereas the nutritional quality of a food choice increased. It was considered that having two separate labels on the food packaging was strenuous in making a healthier as well as a more environmentally sustainable food choice [35]. The Eco-Score does also resemble the Nutri-Score in its appearance (colour and letter). Still, when the Eco-Score was displayed more prominently, similar results were observed [36].

While separate FOP labels may be effectively used for consumer’s food choice according to either nutritional quality or environmental impact, these labels may not be necessarily nor equally effective when positioned as a dual labelling system (i.e. having both separate labels on the packaging). This was also suggested by a study in the United States of America, in which it was observed that the healthiness of a food choice was prioritized over the environmental impact of a food choice [37]. Additionally, from a recent study from Germany, results indicated that consumers may perceive Nutri-Score and Eco-Score differently according to the classifications when both are applied on the packaging, as in some cases a food with an unfavourable Nutri-Score and a favourable Eco-Score was perceived to be more healthy [38]. Therefore, a FOP label which is able to fully reflect both the nutritional quality and the environmental impact of foods may be more effective than a dual labelling system.

The present study was carried out using generic data on food compositions and environmental impact which may be a limitation as these data do not represent the total supply of branded foods. However, as environmental impact data of branded foods are scarce, due to it being labor intensive to obtain these data, it was useful to use generic data on food compositions and environmental impact for exploratory analyses as carried out in the present study. Other limitations are related to the assumptions that had to be applied for the calculation of Nutri-Score classifications, such as the fruits, vegetables and legumes content. Besides, some food groups had relatively smaller sample sizes and extrapolations were applied based on NEVO-code, for which results should be interpreted with caution.

Nutri-Score, as a FOP nutritional label, is found to be successful in discriminating foods based on their nutritional quality [14–17, 19–21]. Consumers may prioritize nutritional quality over environmental impact when using Nutri-Score alongside a FOP environmental label, however, when both of these aspects are well reflected within the Nutri-Score system, the use of Nutri-Score may be beneficial for both human and planetary health. It is therefore recommended to further explore revisions to Nutri-Score or the use of additional tools to guide consumers on both nutritional quality and environmental impact. Further investigations are also recommended to apply data of branded foods.

## Conclusion

Nutri-Score classifications align to a certain extent with GHG emissions and blue water consumption for the general foods, and not for the category of added fats, nuts and seeds. Within groups of general foods, Nutri-Score classifications work to select foods with better nutritional quality (towards Nutri-Score A) as well as lower environmental impacts for a limited number of food groups (e.g. bread and savoury sauces). For most of the other food groups, the conclusion on nutritional quality does not necessarily align with environmental impacts. With the current underlying algorithm, Nutri-Score is not sufficiently able to discriminate foods based on environmental impact as discrimination was limited to only specific general food groups. To use Front-of-Pack labeling to guide consumers on both nutritional quality and environmental impact, exploration of revisions to Nutri-Score or the use of additional tools is needed.

## Electronic supplementary material

Below is the link to the electronic supplementary material.

Supplementary Material 1
