# Supplementary material for: The potential of Nutri-Score to discriminate foods according to environmental impact
Source: Eur J Nutr. 2025 Mar 12;64(3):121. doi: 10.1007/s00394-025-03635-8 (PMC11903517; doi:10.1007/s00394-025-03635-8)
Supplement: Supplementary file 1 — Supplementary Material 1 [file 394_2025_3635_MOESM1_ESM.docx]

**Supplementary Material**

**Journal:** European Journal of Nutrition

**Title:** *The potential of Nutri-Score to discriminate foods according to environmental impact*

**Authors:** Elly Steenbergen^a^, Reina E Vellinga^a^, Joline WJ Beulens^bc^, Elisabeth HM Temme^ad^

^a^National Institute for Public Health and the Environment, Bilthoven, The Netherlands

^b^Department of Epidemiology & Data Science, Amsterdam UMC, location Vrije Universiteit, Amsterdam, The Netherlands

^c^Amsterdam Public Health research institute, Amsterdam, The Netherlands

^d^Wageningen University & Research, Division of Human Nutrition and Health, Wageningen, The Netherlands

**Corresponding author:** Elly Steenbergen, [elly.steenbergen@rivm.nl](mailto:elly.steenbergen@rivm.nl)

**Contents:**

**Supplementary Table S1** Food groups and their assumed fruits, vegetables and legumes content (%) used for the calculations of Nutri-Score

**Supplementary Table S2** Distribution of final score of Nutri-Score of foods by food group

**Supplementary Table S3** Distribution of Nutri-Score classifications (A-E, %) of foods by food group

**Supplementary Table S4** Distribution of environmental impact indicators (greenhouse gas emission and blue water consumption) per kg of food by category of Nutri-Score algorithm

**Supplementary Table S5** Distribution of blue water consumption (m³) per kg of food by food group, in boxplot

**Supplementary Table S6** Spearman’s correlation between final score of Nutri-Score and greenhouse gas emission (kg CO_2_ equivalents) and blue water consumption (m^3^)

**Supplementary Table S7** Median (P50) greenhouse gas emission (kg CO_2_ equivalents) and blue water consumption (m^3^) per kg of foods within Nutri-Score classifications by food group

**Supplementary Figure S1** Distribution of greenhouse gas emissions (kg CO_2_ equivalents) per kg of food by category of Nutri-Score, in boxplot

**Supplementary Figure S2** Distribution of blue water consumption (m³) per kg of food by category of Nutri-Score, in boxplot

**Supplementary Figure S3** Distribution of greenhouse gas emissions (kg CO_2_ equivalents) per kg of food by food group, in boxplot

**Supplementary Figure S4** Distribution of blue water consumption (m³) per kg of food by food group, in boxplot

**Table S1** Food groups and their assumed fruits, vegetables and legumes content (%) used for the calculations of Nutri-Score

| Food group | N | Fruits, vegetables and legumes content (%)* | Exceptions within food group |
| --- | --- | --- | --- |
| Vegetables | 177 | >80 |  |
| Legumes | 24 | >80 |  |
| Fruits | 95 | >80 |  |
| Bread | 111 | ≤40 |  |
| Cereals and cereal products | 84 | ≤40 |  |
| Potatoes and tubers | 28 | ≤40 |  |
| Nuts and seeds | 33 | ≤40 |  |
| Milk and milk products | 113 | ≤40 | Dairy based added fats (e.g. cream) are calculated according to algorithm for added fats, nuts and seeds. Dairy based beverages are calculated according to algorithm for beverages. Dairy based solid foods are calculated according to algorithm for general foods. |
| Meat and dairy substitutes | 43 | >40 | Dairy substitutes of beverages (e.g. soy drinks) are calculated according to algorithm for beverages. Dairy substitutes of added fats (e.g. plant based cream) are calculated according to algorithm for added fats, nuts and seeds. Meat and dairy substitutes of solid foods are calculated according to algorithm for general foods. |
| Fish | 75 | ≤40 |  |
| Cheese | 65 | ≤40 |  |
| Cold meat cuts | 55 | ≤40 | Point allocation for protein differed for red meat. |
| Meat and poultry | 115 | ≤40 | Point allocation for protein differed for red meat. |
| Soups | 25 | ≤40 |  |
| Sugar, sweets and sweet sauces | 115 | ≤40 |  |
| Savoury sauces | 69 | ≤40 |  |
| Pastry and biscuits | 147 | ≤40 |  |
| Savoury snacks | 50 | ≤40 |  |
| Savoury bread spreads | 21 | ≤40 | Spreads based on nuts and seeds (e.g. peanut butter) are calculated according to algorithm for added fats, nuts and seeds |
| Fats and oils | 59 | ≤40 | Fats and oils based on fruits, vegetables and legumes (e.g. olive oil) contain >80% fruits, vegetables and legumes. |
| Non-alcoholic beverages | 107 | ≤40 | Mineral waters always receive Nutri-Score classification A. Beverages which included the terms ‘light’, ‘zero’, ‘sweeteners’ or ‘sugar free’ were considered to contain non-nutritive sweeteners. 100% fruit juices contain >80% fruit, vegetables and legumes content. |

*assumptions based on expert judgement

**Table S2** Distribution of final score of Nutri-Score of foods by food group

|  | Final score of Nutri-Score | | | | | | |
| --- | --- | --- | --- | --- | --- | --- | --- |
| Food group | N | Mean | P5 | P25 | P50 | P75 | P95 |
| Vegetables | 177 | -4 | -8 | -5 | -5 | -4 | 0 |
| Legumes | 24 | -10 | -12 | -12 | -11 | -9 | -5 |
| Fruits | 95 | 0 | -5 | -3 | -2 | 0 | 10 |
| Bread | 111 | 6 | -2 | 0 | 3 | 13 | 21 |
| Cereals and cereal products | 84 | 5 | -6 | -1 | 3 | 11 | 18 |
| Potatoes and tubers | 28 | 2 | 0 | 1 | 2 | 4 | 5 |
| Nuts and seeds | 33 | 0 | -10 | -6 | -2 | 6 | 16 |
| Milk and milk products | 113 | 7 | -1 | 2 | 5 | 12 | 19 |
| Meat and dairy substitutes | 43 | 5 | -4 | -1 | 2 | 11 | 19 |
| Fish | 75 | 3 | -6 | -5 | -1 | 11 | 24 |
| Cheese | 65 | 15 | 7 | 13 | 15 | 17 | 20 |
| Cold meat cuts | 55 | 20 | 12 | 16 | 20 | 23 | 31 |
| Meat and poultry | 115 | 5 | -5 | -1 | 2 | 11 | 23 |
| Soups | 25 | 3 | 1 | 2 | 3 | 4 | 5 |
| Sugar, sweets and sweet sauces | 115 | 22 | 6 | 18 | 20 | 28 | 32 |
| Savoury sauces | 69 | 13 | 1 | 8 | 13 | 18 | 23 |
| Pastry and biscuits | 147 | 20 | 8 | 15 | 21 | 26 | 29 |
| Savoury snacks | 50 | 16 | 3 | 11 | 17 | 21 | 24 |
| Savoury bread spreads | 21 | 10 | 2 | 8 | 11 | 14 | 17 |
| Fats and oils | 59 | 9 | 1 | 4 | 7 | 16 | 24 |
| Non-alcoholic beverages | 107 | 8 | 0 | 4 | 7 | 12 | 20 |

**Table S3** Distribution of Nutri-Score classifications (A-E, %) of foods by food group

|  |  | Nutri-Score classification (%) | | | | |
| --- | --- | --- | --- | --- | --- | --- |
| Food group | N | A | B | C | D | E |
| Vegetables | 177 | 95 | 2 | 0 | 2 | 1 |
| Legumes | 24 | 100 | 0 | 0 | 0 | 0 |
| Fruits | 95 | 76 | 4 | 16 | 4 | 0 |
| Bread | 111 | 37 | 8 | 23 | 23 | 9 |
| Cereals and cereal products | 84 | 36 | 13 | 19 | 29 | 4 |
| Potatoes and tubers | 28 | 14 | 54 | 32 | 0 | 0 |
| Nuts and seeds | 33 | 36 | 24 | 30 | 6 | 3 |
| Milk and milk products | 113 | 12 | 19 | 35 | 19 | 16 |
| Meat and dairy substitutes | 43 | 19 | 33 | 21 | 21 | 7 |
| Fish | 75 | 59 | 11 | 4 | 17 | 9 |
| Cheese | 65 | 0 | 0 | 11 | 74 | 15 |
| Cold meat cuts | 55 | 0 | 0 | 0 | 35 | 65 |
| Meat and poultry | 115 | 37 | 16 | 23 | 17 | 9 |
| Soups | 25 | 0 | 32 | 68 | 0 | 0 |
| Sugar, sweets and sweet sauces | 115 | 0 | 3 | 5 | 18 | 74 |
| Savoury sauces | 69 | 4 | 1 | 28 | 48 | 19 |
| Pastry and biscuits | 147 | 0 | 0 | 8 | 32 | 60 |
| Savoury snacks | 50 | 2 | 2 | 14 | 42 | 40 |
| Savoury bread spreads | 21 | 5 | 14 | 19 | 57 | 5 |
| Fats and oils | 59 | 0 | 8 | 63 | 15 | 14 |
| Non-alcoholic beverages | 107 | 2 | 15 | 30 | 14 | 39 |

**Table S4** Distribution of environmental impact indicators (greenhouse gas emission and blue water consumption) per kg of food by category of Nutri-Score algorithm

|  | Greenhouse gas emissions (kg CO_2_ equivalents) | | | | | | | Blue water consumption (m^3^) | | | | | |
| --- | --- | --- | --- | --- | --- | --- | --- | --- | --- | --- | --- | --- | --- |
| Category of algorithm | N | Mean | P5 | P25 | P50 | P75 | P95 | Mean | P5 | P25 | P50 | P75 | P95 |
| General foods | 1341 | 5.15 | 0.72 | 1.40 | 2.51 | 6.06 | 15.4 | 0.11 | 0.01 | 0.02 | 0.05 | 0.11 | 0.36 |
| Added fats, nuts and seeds | 118 | 4.81 | 1.54 | 2.52 | 4.46 | 5.01 | 12.2 | 0.63 | 0.02 | 0.05 | 0.10 | 0.17 | 4.09 |
| Beverages | 152 | 1.81 | 0.32 | 0.62 | 0.93 | 2.19 | 3.61 | 0.07 | 0.01 | 0.01 | 0.02 | 0.09 | 0.33 |

**Table S5** Spearman’s correlation between algorithm component points and greenhouse gas emission (kg CO_2_ equivalents) and blue water consumption (m^3^)

| Food group | Algorithm component |  |  | GHGe | BWC |
| --- | --- | --- | --- | --- | --- |
| General foods | Energy component |  | r_s_ | 0.22 | -0.03 |
|  |  |  | p | 0.000 | 0.347 |
|  | Sugar component |  | r_s_ | -0.23 | -0.05 |
|  |  |  | p | 0.000 | 0.044 |
|  | SFA component |  | r_s_ | 0.52 | 0.13 |
|  |  |  | p | 0.000 | 0.000 |
|  | Salt component |  | r_s_ | 0.33 | 0.06 |
|  |  |  | p | 0.000 | 0.031 |
|  | Fiber component |  | r_s_ | -0.22 | -0.08 |
|  |  |  | p | 0.000 | 0.006 |
|  | Protein component |  | r_s_ | 0.43 | 0.03 |
|  |  |  | p | 0.000 | 0.204 |
| Added fats, nuts and seeds | Energy component |  | r_s_ | 0.58 | -0.04 |
|  |  |  | p | 0.000 | 0.635 |
|  | Sugar component |  | r_s_ | -0.08 | 0.10 |
|  |  |  | p | 0.398 | 0.287 |
|  | SFA component |  | r_s_ | 0.00 | 0.07 |
|  |  |  | p | 0.998 | 0.000 |
|  | Salt component |  | r_s_ | 0.07 | 0.03 |
|  |  |  | p | 0.431 | 0.780 |
|  | Fiber component |  | r_s_ | -0.06 | 0.70 |
|  |  |  | p | 0.530 | 0.000 |
|  | Protein component |  | r_s_ | -0.20 | 0.51 |
|  |  |  | p | 0.028 | 0.000 |
| Beverages | Energy component |  | r_s_ | 0.54 | 0.27 |
|  |  |  | p | 0.000 | 0.001 |
|  | Sugar component |  | r_s_ | 0.34 | 0.34 |
|  |  |  | p | 0.000 | 0.000 |
|  | SFA component |  | r_s_ | 0.34 | 0.02 |
|  |  |  | p | 0.000 | 0.854 |
|  | Salt component |  | r_s_ | 0.40 | 0.31 |
|  |  |  | p | 0.000 | 0.000 |
|  | Fiber component |  | r_s_ | 0.21 | 0.14 |
|  |  |  | p | 0.081 | 0.000 |
|  | Protein component |  | r_s_ | 0.58 | -0.09 |
|  |  |  | p | 0.000 | 0.204 |

GHGe: greenhouse gas emission (kg CO_2_ equivalents), BWC: blue water consumption (m^3^), r_s_: Spearman’s correlation coefficient, *p*: p-value

**Table S6** Spearman’s correlation between final score of Nutri-Score and greenhouse gas emission (kg CO_2_ equivalents) and blue water consumption (m^3^)

| Food group | N |  | GHGe | BWC |
| --- | --- | --- | --- | --- |
| Vegetables | 177 | r_s_ | 0.14 | 0.18 |
|  |  | *p* | *0.056* | *0.014* |
| Legumes | 24 | r_s_ | -0.26 | 0.44 |
|  |  | *p* | *0.228* | *0.030* |
| Fruits | 95 | r_s_ | 0.20 | 0.17 |
|  |  | *p* | *0.054* | *0.093* |
| Bread | 111 | r_s_ | 0.60 | 0.53 |
|  |  | *p* | *0.000* | *0.000* |
| Cereals and cereal products | 84 | r_s_ | 0.46 | 0.39 |
|  |  | *p* | *0.000* | *0.000* |
| Potatoes and tubers | 28 | r_s_ | 0.36 | 0.39 |
|  |  | *p* | *0.058* | *0.038* |
| Nuts and seeds | 33 | r_s_ | 0.57 | -0.15 |
|  |  | *p* | *0.001* | *0.391* |
| Milk and milk products | 113 | r_s_ | 0.35 | 0.40 |
|  |  | *p* | *0.000* | *0.000* |
| Meat and dairy substitutes | 43 | r_s_ | 0.05 | 0.15 |
|  |  | *p* | *0.744* | *0.333* |
| Fish | 75 | r_s_ | -0.48 | -0.44 |
|  |  | *p* | *0.000* | *0.000* |
| Cheese | 65 | r_s_ | 0.13 | 0.28 |
|  |  | *p* | *0.291* | *0.025* |
| Cold meat cuts | 55 | r_s_ | 0.14 | 0.09 |
|  |  | *p* | *0.300* | *0.498* |
| Meat and poultry | 115 | r_s_ | 0.01 | -0.26 |
|  |  | *p* | *0.914* | *0.005* |
| Soups | 25 | r_s_ | -0.63 | -0.65 |
|  |  | *p* | *0.001* | *0.000* |
| Sugar, sweets and sweet sauces | 115 | r_s_ | 0.58 | 0.32 |
|  |  | *p* | *0.000* | *0.000* |
| Savoury sauces | 69 | r_s_ | 0.52 | 0.53 |
|  |  | *p* | *0.000* | *0.000* |
| Pastry and biscuits | 147 | r_s_ | 0.51 | -0.01 |
|  |  | *p* | *0.000* | *0.923* |
| Savoury snacks | 50 | r_s_ | 0.19 | -0.18 |
|  |  | *p* | *0.197* | *0.213* |
| Savoury bread spreads | 21 | r_s_ | -0.12 | -0.41 |
|  |  | *p* | *0.619* | *0.063* |
| Fats and oils | 59 | r_s_ | 0.38 | -0.14 |
|  |  | *p* | *0.003* | *0.282* |
| Non-alcoholic beverages | 107 | r_s_ | 0.19 | 0.06 |
|  |  | *p* | *0.051* | *0.548* |

GHGe: greenhouse gas emission (kg CO_2_ equivalents), BWC: blue water consumption (m^3^), r_s_: Spearman’s correlation coefficient, *p*: p-value

**Table S7** Median (P50) greenhouse gas emission (kg CO_2_ equivalents) and blue water consumption (m^3^) per kg of foods within Nutri-Score classifications by food group

|  | Median greenhouse gas emission (kg CO_2_ equivalents) within Nutri-Score classifications | | | | | | | | | | Median blue water consumption (m^3^) within Nutri-Score classifications | | | | | | | | | |
| --- | --- | --- | --- | --- | --- | --- | --- | --- | --- | --- | --- | --- | --- | --- | --- | --- | --- | --- | --- | --- |
|  | A | | B | | C | | D | | E | | A | | B | | C | | D | | E | |
| Food group | N | P50 | N | P50 | N | P50 | N | P50 | N | P50 | N | P50 | N | P50 | N | P50 | N | P50 | N | P50 |
| Vegetables | 169 | 1.56 | 4 | 1.62 | . | . | 3 | 2.34 | 1 | 6.63 | 169 | 0.05 | 4 | 0.06 | . | . | 3 | 0.11 | 1 | 0.43 |
| Legumes | 24 | 1.93 | . | . | . | . | . | . | . | . | 24 | 0.07 | . | . | . | . | . | . | . | . |
| Fruits | 72 | 1.13 | 4 | 1.32 | 15 | 2.44 | 4 | 3.54 | . | . | 72 | 0.20 | 4 | 0.37 | 15 | 0.70 | 4 | 0.60 | . | . |
| Bread | 41 | 1.19 | 9 | 1.19 | 25 | 1.23 | 26 | 1.77 | 10 | 2.42 | 41 | 0.02 | 9 | 0.02 | 25 | 0.02 | 26 | 0.06 | 10 | 0.04 |
| Cereals and cereal |  |  |  |  |  |  |  |  |  |  |  |  |  |  |  |  |  |  |  |  |
| products | 30 | 1.05 | 11 | 1.63 | 16 | 1.55 | 24 | 1.71 | 3 | 2.42 | 30 | 0.02 | 11 | 0.03 | 16 | 0.02 | 24 | 0.06 | 3 | 0.04 |
| Potatoes and tubers | 4 | 1.01 | 15 | 1.09 | 9 | 6.15 | . | . | . | . | 4 | 0.01 | 15 | 0.01 | 9 | 0.09 | . | . | . | . |
| Nuts and seeds | 12 | 3.60 | 8 | 4.06 | 10 | 4.33 | 2 | 5.73 | 1 | 4.91 | 12 | 0.86 | 8 | 3.31 | 10 | 1.90 | 2 | 0.11 | 1 | 0.07 |
| Milk and milk products | 13 | 2.30 | 22 | 2.03 | 39 | 2.19 | 21 | 2.41 | 18 | 2.47 | 13 | 0.02 | 22 | 0.02 | 39 | 0.02 | 21 | 0.02 | 18 | 0.02 |
| Meat and dairy substitutes | 8 | 4.39 | 14 | 0.76 | 9 | 2.95 | 9 | 3.77 | 3 | 2.92 | 8 | 0.07 | 14 | 0.01 | 9 | 0.04 | 9 | 0.04 | 3 | 0.06 |
| Fish | 44 | 7.26 | 8 | 6.75 | 3 | 6.95 | 13 | 2.81 | 7 | 2.81 | 44 | 0.04 | 8 | 0.04 | 3 | 0.04 | 13 | 0.02 | 7 | 0.02 |
| Cheese | . | . | . | . | 7 | 8.80 | 48 | 10.72 | 10 | 9.90 | . | . | . | . | 7 | 0.07 | 48 | 0.09 | 10 | 0.10 |
| Cold meat cuts | . | . | . | . | . | . | 19 | 10.82 | 36 | 11.98 | . | . | . | . | . | . | 19 | 0.10 | 36 | 0.11 |
| Meat and poultry | 42 | 12.42 | 18 | 30.28 | 26 | 25.20 | 19 | 15.68 | 10 | 9.76 | 42 | 0.15 | 18 | 0.24 | 26 | 0.20 | 19 | 0.17 | 10 | 0.10 |
| Soups | . | . | 8 | 2.82 | 17 | 0.71 | . | . | . | . | . | . | 8 | 0.05 | 17 | 0.03 | . | . | . | . |
| Sugar, sweets and |  |  |  |  |  |  |  |  |  |  |  |  |  |  |  |  |  |  |  |  |
| sweet sauces | . | . | 3 | 1.13 | 6 | 1.10 | 21 | 1.47 | 85 | 2.70 | . | . | 3 | 0.01 | 6 | 0.03 | 21 | 0.06 | 85 | 0.03 |
| Savoury sauces | 3 | 0.43 | 1 | 2.05 | 19 | 1.72 | 33 | 2.92 | 13 | 4.33 | 3 | 0.01 | 1 | 0.02 | 19 | 0.04 | 33 | 0.04 | 13 | 0.07 |
| Pastry and biscuits | . | . | . | . | 12 | 2.29 | 47 | 2.72 | 88 | 3.86 | . | . | . | . | 12 | 0.06 | 47 | 0.04 | 88 | 0.06 |
| Savoury snacks | 1 | 0.95 | 1 | 1.07 | 7 | 3.47 | 21 | 4.83 | 20 | 4.83 | 1 | 0.09 | 1 | 0.01 | 7 | 0.05 | 21 | 0.08 | 20 | 0.06 |


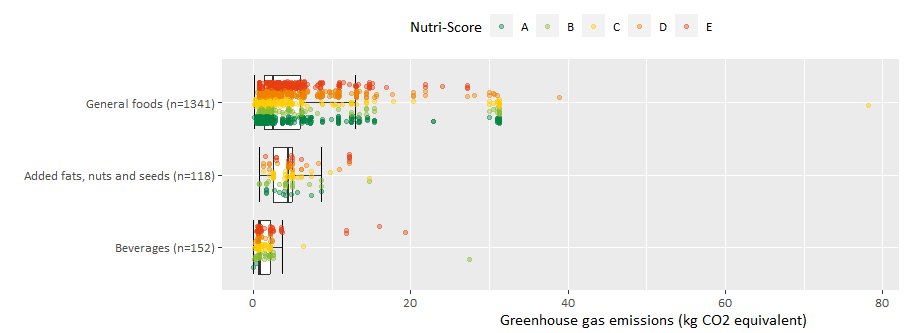


**Fig. S1** Distribution of greenhouse gas emissions (kg CO_2_ equivalents) per kg of food by category of Nutri-Score, in boxplot. Dots reflect individual observations according to the colours of Nutri-Score classifications A-E. The white box represents the interquartile range, the black vertical line in the box represents the median of the data, the whiskers of the box present the minimum and maximum values of the data, excluding the outliers. The specific outlier within general foods for greenhouse gas emissions is dried goji berries


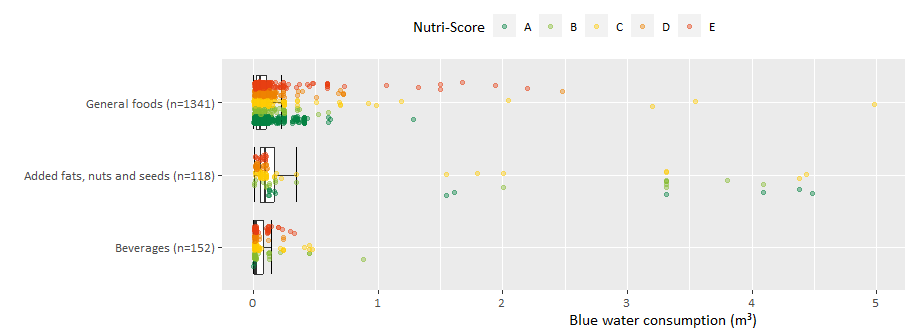
**Fig. S2** Distribution of blue water consumption (m³) per kg of food by category of Nutri-Score, in boxplot. Dots reflect individual observations according to the colours of Nutri-Score classifications A-E. The white box represents the interquartile range, the black vertical line in the box represents the median of the data, the whiskers of the box present the minimum and maximum values of the data, excluding the outliers


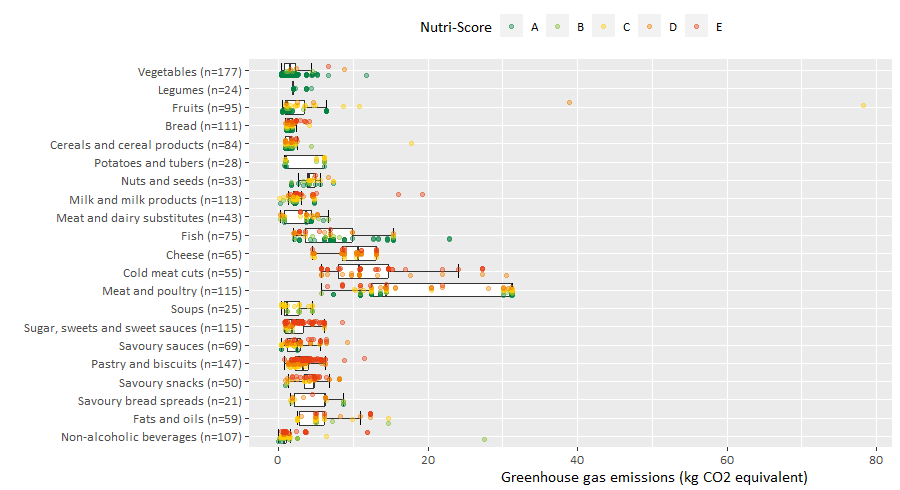


**Fig. S3** Distribution of greenhouse gas emissions (kg CO_2_ equivalents) per kg of food by food group, in boxplot. Dots reflect individual observations according to the colours of Nutri-Score classifications A-E. The white box represents the interquartile range, the black vertical line in the box represents the median of the data, the whiskers of the box present the minimum and maximum values of the data, excluding the outliers. The specific outlier within general foods for greenhouse gas emissions is dried goji berries


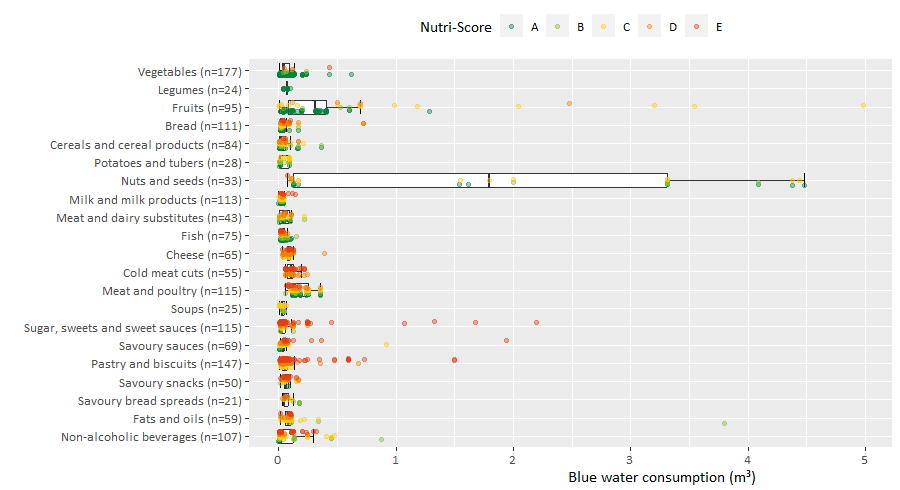


**Fig. S4** Distribution of blue water consumption (m³) per kg of food by food group, in boxplot. Dots reflect individual observations according to the colours of Nutri-Score classifications A-E. The white box represents the interquartile range, the black vertical line in the box represents the median of the data, the whiskers of the box present the minimum and maximum values of the data, excluding the outliers
